# Comparison of high-pressure, freeze-thaw cycles and germination-parboiling treatments on lipids digestibility and rancidity of brown rice

**DOI:** 10.1038/s41598-022-19272-8

**Published:** 2022-09-19

**Authors:** Hao Wang, Qiang Wang, Jiahong Zhu, Guixian Hu

**Affiliations:** Institute of Agro-Product Safety and Nutrition, Zhejiang Academy of Agricultural Sciences, 298 Deshengzhong Road, Hangzhou, 310021 China

**Keywords:** Fatty acids, Lipid peroxides

## Abstract

Brown rice (BR) contains more lipids, as compared with white rice, but its indigestibility and rancidity limit the consumer acceptability. Therefore, the objective of this study was to compare the effect of high-pressure (HP), freeze–thaw cycles (FTC) and germination-parboiling (GP) treatments on lipid in vitro digestibility and rancidity of BR. GP treated BR released the most fatty acids (especially palmitic acid and stearic acid) after in vitro digestion, followed by FTC treated BR. FTC treated BR kept the highest value of fat acidity during storage, while opposite results were observed in GP treated BR. Although HP treatment increased fat acidity value immediately, it slowed down the increase of fat acidity with storage. The results of conjugated dienes and malonaldehydes content in BR stored under accelerated conditions indicated better lipid oxidation stability after HP and GP treatment, and that it’s necessary for FTC treated BR products to be stored under anaerobic conditions.

## Introduction

Brown rice (BR) is a whole grain and consists of endosperm (90% of its total weight), embryo (2–3%) and bran layers (6–7%)^1^. To get soft texture and taste, BR is usually processed into white rice (WR) by removal of embryo and bran layers, which contains various nutrients including lipids. Lipids are the third major component of BR, following starch and protein. The lipid content of BR is almost four times higher than that of WR, and these lipids make a significant contribution to processing and nutritional properties. The essential fatty acids in BR lipids account for 36.9–39.1%^2^. Considering the fatty acids composition, BR could be a good source of healthy fatty acids for consumers in daily diet^3^.

However, compared with WR, BR is more resistant to digestion because rice bran contains 7–11% indigestible crude fiber, while endosperm contains only 0.2–0.5%^4^. Bran layers inhibit the absorption of moisture and gastric acid, delaying the rice disintegration and dissolution. After digestion, the particles of cooked WR fractions are smaller than those of cooked BR fractions^5^. Poor digestibility results in underutilization of the nutrients in BR. In previous research, it was proved that high-pressure (HP), freeze–thaw cycles (FTC) and germination-parboiling (GP) treatments could improve the release of glucose, amino acids, total phenolics, flavonoid and minerals after BR digestion, basing on the structural destruction and effects on properties of nutrients^1,6–8^, and these processing technologies have been applied to produce commercial BR products. However, related research has not worked on improving lipids digestibility of whole BR.

Due to higher lipid content, BR is also more susceptible to rancidity during storage, which also limits its consumer acceptability. As a result of BR rancidity, lipids are hydrolyzed into free fatty acids, and/or oxidized into volatile short-chain aldehydes and ketones, causing off-flavor. Several conventional technologies have been applied for inhibiting rancidity of BR. Heat treatment reduced lipid hydrolysis during storage due to inactivation of lipase and lipoxidase^9^. Li et al.^10^ pointed out that a combination of selenium enrichment and germination could delay oxidative rancidity. Besides, the effects of innovative processing technologies of BR on rancidity are also the focus of current research. Wang et al.^11^ found that HP treatment at 200 MPa was efficient to reduce rancidity in BR during storage, while HP treatment at higher pressure levels enhanced lipid hydrolysis. Although FTC treatment could effectively improve texture and protein digestibility of BR^1,6^, its effect on BR rancidity has not been studied.

The objective of this study was therefore to compare the effects of HP, FTC, and GP treatments on lipid digestibility and rancidity of BR. Hydrolysis and oxidation of lipids in BR were analyzed to understand the changes induced by these three treatments. Such a study will provide a point of reference for industrial production, storage, and consumption.

## Materials and methods

### Materials

Commercial-grade hybrid rice (variety Chuanyou 623) was obtained as brown rice (long grain, 3.7% amylose content, 10.9% moisture content) from Sichuan Danonghe Agricultural Development Co. Ltd. in Sichuan Province, China. The brown rice was vacuum-sealed in a polyethylene pouch with a desiccant and stored at 4 °C. Untreated BR was considered control for comparison with the treated samples.

### Samples preparation

All treatments were based on the protocols established in previous study^1^ and are described below.

#### High-pressure treatment

The HP treatment was performed in a laboratory-scale high-pressure chamber (UHPF-750, Kefa, Baotou, China). Water was used as the pressure transmission medium. The pressure increase rate was about 150 MPa/min, and the depressurization time was less than 10 s. The pressure increase and depressurization times were not included in the HP treatment time. For HP treatment, 10 g samples of rice kernels were first vacuum-sealed in polyethylene pouches (6 cm × 8.5 cm) together with 16 mL of deionized water and incubated at 25 °C for 3 h in a constant-temperature water bath (DKS, Zhongxin, Jiaxing, China). These pouches were then transferred to the high-pressure chamber and equilibrated to 20 °C before pressurization. Two HP treatments were used in this study, and both were performed at 200 MPa. The first treatment involved a single cycle with a post-pressurized holding time of 10 min. The second treatment was a two-cycle pressure treatment with a holding time of 5 min in each cycle, and no wait period between the pressure cycles. Thus, the two HP treatments, designated HP-10 min and HP-5 + 5 min, both had a total holding time of 10 min but involved depressurization and repressurization.

#### Freeze–thaw cycle treatment

For FTC treatment, 10 g samples of rice kernels were packed in polyethylene pouches (6 cm × 8.5 cm) together with 10 mL of deionized water and soaked at 25 °C for 60 min in a constant-temperature water bath. The soaked grains were then drained and vacuum sealed in a fresh polyethylene pouch (6 cm × 8.5 cm). For each treatment, the samples were first frozen at −20 °C for 1 h in a freezer and then immediately thawed at 35 °C for 40 min in a constant-temperature water bath The FTC treatments were repeated for two cycles (FTC-2) or four cycles (FTC-4).

#### Germination-parboiling treatment

For GP treatment, samples (10 g) of BR were germinated in the dark with 30 mL of deionized water at 30 °C for 12 or 24 h (designated G12P and G24P). Following the germination process, the soak water was drained out, and the BR was dried at 30 °C for 40 min to adjust the moisture content to ~ 30% (wet basis). The samples were then steamed for 10 min (parboiling) in a rice cooker (CFXB30-90, Triangle, Guangzhou, China) to pre-gelatinize the starch. For steaming, the rice kernels were placed in a metal tray with a lid, and after the water started to boil in the rice cooker, the tray was placed 15 cm above the boiling water level (representing atmospheric pressure steam cooking at 98–100 °C) for 10 min.

All treated samples were subsequently dried in an oven (D6X-9073, FM, Shanghai, China) at 30–35 °C until the average moisture content was about 11 ± 0.5% (wet basis). All experiments were conducted in triplicate.

### In vitro digestion

The rice samples were cooked in individual aluminum containers (9 cm × 3.5 cm × 2.5 cm) (10 g rice in 16 mL of distilled water) using a steam cooker (JBA-S18C, Tiger, Osaka, Japan) for 40 min, and then the cooked rice was cooled to room temperature and freeze-dried (Scientz-10 N, Xinzhi, Ningbo, China). The freeze-dried cooked rice was ground in a laboratory pulverizer (DFY-200, Lingda Machinery, Wenling, China) into moderately fine particles. The ground samples that passed through 600 μm but were retained on 400 μm (rice grits) were used for the in vitro digestion test. This particle size range was selected to minimize the deviation arising from differences in particle size or grain fragments and to mimic the size of chewed cooked rice during mastication^12^.

A simulated in vitro digestion technique was used based on the method suggested by Miller et al.^13^ with some modifications. Four grams of ground rice fragments were mixed with distilled water (40 mL). For gastric digestion, 1 M HCl (about 0.4 mL) was added to the rice-water mixture to adjust the pH to 2.0, followed by 2 mL of pepsin solution, which consisted of 1 g of pepsin (P7000, Sigma, Madrid, Spain) dissolved in 100 mL of 0.01 M HCl. The sample was agitated in a shaker incubator (HZ-9211 KB, Hualida, Taicang, China) at 37 °C for 2 h. For intestinal digestion, the pH of the solution was adjusted to 7.0 using 1 M NaHCO_3_, and then 1 mL of pancreatin bile solution was added, which consisted of 0.4 g of pancreatin (93,615, Sigma, Madrid, Spain) and 2.5 of g of bile extract (SXBK Biotech Co. Ltd., Xian, China) dissolved in 100 mL of 0.1 M NaHCO_3_. The mixture was again shaken in the shaker incubator at 37 °C for 2 h. The digested solution was heated in a boiling water bath for 10 min to inactivate the enzymes, followed by cooling and centrifugation for 20 min at 10,000 × g (5810R, Eppendorf AG, Hamburg, Germany). The supernatant was collected for subsequent analyses of released free fatty acids.

### Gas chromatographic analysis of released free fatty acids

Released free fatty acids in the supernatant were determined according to the method described by Rodrigues et al.^14^ with some modifications. Ten mL of the supernatant was heated to evaporate the water. The residue was mixed with 1 mL of boron trifluoride-methanol solution (B822338, Macklin, Shanghai, China) and heated at 70 °C for 1 h for methyl esterification. After methyl esterification, the solution was fully mixed with 1 mL of saturated NaCl solution and 1 mL of n-hexane, and then was left standing and stratified. Fatty acid methyl ester (FAME) content in n-hexane layer was determined by gas chromatography (7890A, Agilent, Santa Clara, CA, USA) equipped with a flame ionization detector (FID). 0.2 μL of FAME solution was injected and analysis were performed with a 60 m × 0.25 mm i.d. × 0.25 µm fused silica capillary column (DB-23, Agilent, Santa Clara, CA, USA). The initial temperature of 120 °C was maintained for 5 min, then raised to 190 °C at a rate of 5 °C/min, maintained for 12 min, and then increased to 210 °C at a rate of 2.5 °C/min, maintained for 10 min. The split ratio was 1:10, and nitrogen was the carrier gas at a rate of 1 mL/min. The injector and detector temperatures were 270 °C and 280 °C, respectively.

### Rancidity of brown rice

To accelerate rancidity, all BR samples were directly stored at 37 °C and 58% relative humidity in temperature controlled incubators (SPX-450, Saifu, Ningbo, China) for 14 days. During storage, samples were selected and analyzed every week.

#### Milling brown rice

All stored BR was milled with laboratory pulverizer and passed through a 100-mesh sieve. Samples were stored at − 20 °C until analysis.

#### Fat acidity

Fat acidity was determined according to ISO 7305:1998^15^ with some modifications. The powdered test sample weighing 1 g (exact to 0.0001 g) was mixed with 10 mL of 95% alcohol and stirred for 1 h at 25 °C. The sample was then centrifuged for 5 min at 2057 g, 25 °C. One mL of supernatant was mixed with 10 mL of 95% alcohol and the solution was titrated with 0.01 mol/ L sodium hydroxide (NaOH), using a microburette (graduated in 0.01 mL divisions). Phenolphthalein was used as indicator, and titration stopped when a pale pink color lasted for 30 s. As a blank test was done with 1 mL of 95% alcohol instead of 1 mL of supernatant. The fat acidity (A_*K*_) was expressed in milligrams of potassium hydroxide (KOH) required to neutralize the fatty acids in 100 g d.w. of BR, which was calculated according to the following Eq. (1):

1 $$A_{K} = \frac{{56100 \times (V_{1} - V_{0} ) \times c}}{m} \times \frac{100}{{100 - w}}$$

where *c* is the exact concentration (mol/L) of NaOH used for titration, *m* is the mass (g) of powder, *V*_1_ is the volume (mL) of the NaOH used for titrating the supernatant, *V*_0_ is the volume (mL) of the NaOH used for titrating the blank, *w* is the moisture content (%) (measured using the method of the ISO 712:2009^16^), 56,100 is a constant to be applied for potassium hydroxide, i.e. (56.1 × 10 × 100).

#### Conjugated dienes (CD) content

CD content was measured based on the method described by Verardo, et al.^17^ with some modifications. Again, powdered sample weighing 1 g (exact to 0.0001 g) was mixed with 5 mL of petroleum ether and shaken (150 rpm) at 20 °C for 24 h. The samples were centrifuged for 5 min at 2057 g, 25 °C. 1.5 mL of supernatant was put into tube to evaporate petroleum ether by slightly heating, after which the samples were diluted to 10 mL with 2,2,4-trimethylpentane. The absorbance of the solution at 233 nm was measured with spectrophotometer (Cary 60 UV/Vis, Agilent, Santa Clara, CA, USA). As a blank, 1.5 mL of petroleum ether was used instead of 1.5 mL of supernatant. CD content was calculated according to the Beer–Lambert law, using an extinction coefficient of 2.525 × 10^4^/M/ cm at 233 nm^18^.

#### 2-Thiobarbituric acid (TBA) value

The TBA value of brown rice was measured based on the spectrophotometry (at 532 nm) of thiobarbituric acid reactive substances. Powdered sample weighing 1 g (exact to 0.0001 g) was mixed with 5 mL of trichloroacetic acid solution (7.5% trichloroacetic acid; 0.1% ethylenediamine tetraacetic acid disodium), stirred for 30 min at 50 °C, and then centrifuged at 5000 g for 10 min. The malonaldehydes (MDA) concentration of supernatant was measured using MDA assay kit purchased from Nanjing Jiancheng Bioengineering Institute (Nanjing, China). Finally, the TBA value was expressed as MDA content (nmol/g of brown rice) according to the following Eq. (2):

2 $${\text{TBA value}} = \frac{V \times c}{m} \times \frac{100}{{100 - w}}$$

where *c* is the MDA concentration (nmol/mL) of supernatant, *m* is the mass (g) of powder, *V* is the volume (mL) of the supernatant, *w* is the moisture content (%).

### Statistical analysis

Statistical analyses were performed using one-way analysis of variance (ANOVA). Duncan’s test (*p* < 0.05) was applied to compare the differences in means using SPSS 20.0 (SPSS Inc., Chicago, Ill, USA).

## Results

### In vitro digestion

Palmitic acid (C16:0), stearic acid (C18:0), oleic acid (C18:1) and linoleic acid (C18:2) were in the majority of the total released fatty acids and were listed in Table 1, so other fatty acids were not shown. The control released 29.8 μg/g of C18:0, which was considerably lower than all treated BR. Similar results were also observed in released C16:0. Among the treated BR, GP treated BR released the most saturated fatty acids (SFA), especially G12P (321.1 μg/g, including C16:0 and C18:0), followed by FTC treated BR. Total released SFA was 41% higher for FTC-4 than for FTC-2. HP treatments showed the slighter improvement on the total release of two SFA, only 29.9–48.9 μg/g more than the control.

**Table 1:** Main released fatty acids (μg/g) of brown rice processed with different treatments after in vitro digestion.

Samples	Released fatty acids			
	C16:0	C18:0	C18:1	C18:2
Control	97.8 ± 9.0^d^	29.8 ± 3.2^f^	14.0 ± 3.2^c^	23.6 ± 2.2^b^
HP-10 min	119.1 ± 16.5^cd^	57.4 ± 8.0^d^	NT	NT
HP-5 + 5 min	112.5 ± 13.5^cd^	45.0 ± 5.2^e^	11.3 ± 1.5^cd^	18.0 ± 1.9^d^
FTC-2	130.0 ± 6.9^c^	76.7 ± 2.2^c^	8.0 ± 0.3^d^	11.1 ± 0.8^e^
FTC-4	172.6 ± 7.2^b^	118.6 ± 5.5^a^	11.2 ± 0.4^cd^	18.7 ± 0.7^cd^
G12P	220.3 ± 18.2^a^	100.8 ± 6.6^b^	33.1 ± 2.0^a^	37.9 ± 2.4^a^
G24P	187.0 ± 25.6^b^	83.8 ± 10.8^c^	18.7 ± 3.0^b^	22.0 ± 3.3^bc^

Values are means ± standard deviations (n = 3). Within columns, means followed by different letters are significantly different (*p* < 0.05).

NT: not detected.

The results of the two unsaturated fatty acids (USFA) (C18:1 and C18:2) were different from the other two SFA. HP treatment at 200 MPa for 5 + 5 min decreased the release of C18:1 and C18:2 by 19% and 24% respectively, which were not detected in digestion solution of HP-10 min. FTC-4 also showed similar decrease in released USFA as HP-5 + 5 min, and FTC-2 showed less release of C18:1 and C18:2, only 57% and 47% of the control. In contrast to HP and FTC treatment, GP treatment increased the amount of released USFA. It was noticed that the GP induced increase of two USFA was much less than that of two SFA.

Linoleic acid and α-linolenic acid are essential fatty acids that cannot be synthesized in the body and are obtained by diet. In this study, only linoleic acid was detected in the digestion solution of all BR samples, and G12P could provide the most essential fatty acids (C18:2, 37.9 μg/g) after digestion. To sum up, GP treated BR (especially G12P) showed the largest amount of fatty acids released after digestion, and were considered as a better choice for consumers to supplement the intake of fatty acids.

### Fat acidity

**Figure 1:**
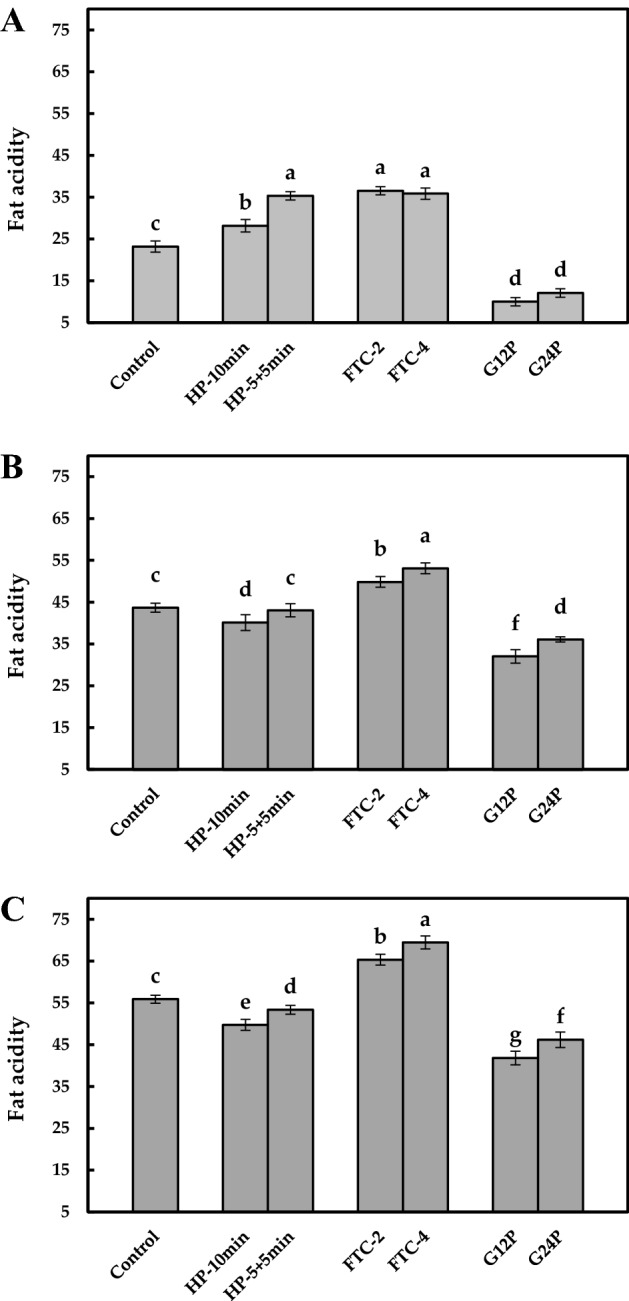
Fat acidity (mg KOH/100 g) of brown rice processed with different treatments after (**A**), 0 day; (**B**), 7 days; (**C**), 14 days of storage. Values are means ± standard deviations (n = 3). Different letters indicate significant differences (*p* < 0.05).

Hydrolytic rancidity of lipids results in the formation of free fatty acids, and are usually characterized by fat acidity. As shown in Fig. 1, the fat acidity of control was 23.2 mg KOH/100 g, which increased to 28.2 mg KOH/100 g (HP-10 min) and 35.3 mg KOH/100 g (HP-5 + 5 min) after HP treatment. Similar with HP-5 + 5 min, FTC-2 and FTC-4 also showed the highest value of fat acidity, 55–57% more than the control. HP and FTC treatments both enhanced hydrolysis of lipids immediately after treatment. In contrast, GP treatment decreased fat acidity value from 23.2 mg KOH/100 g (control) to 10.0 mg KOH/100 g (G12P) and 12.1 mg KOH/100 g (G24P), meaning the degradation of free fatty acids.

After storage, the fat acidity of control increased to 43.7 mg KOH/100 g (7 days) and 55.9 mg KOH/100 g (14 days). The fat acidity of HP-10 min and HP-5 + 5 min increased by only 21.5 mg KOH/100 g and 18.1 mg KOH/100 g respectively after 14 days, substantially lower than control, while FTC treated BR consistently had the highest fat acidity. In particular, FTC-4 accumulated more free fatty acids than FTC-2, up to 69.5 mg KOH/100 g after 14 days. Stored for 7 days, GP treated BR showed substantially increased fat acidity value (32.0–36.1 mg KOH/100 g), which continuously increased to 41.8–46.2 mg KOH/100 g after an additional 7 days of storage. These results indicated that the BR treated with GP treatment contained substantially less free fatty acids than control and the BR treated with other two treatments during 14 days. After 14 days of accelerated storage, the fat acidity values of all BR samples had exceeded the allowable value (37.0 mg KOH/100 g)^19^.

### CD content

In the primary stage of lipid oxidation, USFA are oxidized to form CD, of which the content reflects the degree of primary lipid oxidation. Table 2 summarized the CD content of all BR samples. Before treatments, BR contained 90.5 μmol/100 g of CD, and there was no significant change in CD content of BR after FTC treatment. Similarly, HP treated BR showed no significant difference in CD content from FTC treated BR, but HP-10 min contained substantially more CD than HP-5 + 5 min. It indicated that HP treatment with the mode of continuous 10 min of holding time could cause severer primary peroxidation of lipids (especially USFA). GP treatment substantially promoted the CD content of BR to 96.6 μmol/100 g (G12P) and 98.7 μmol/100 g (G24P).

**Table 2:** Conjugated diene content (μmol/100 g) of brown rice processed with different treatments during storage.

Samples	Storage		
	0 day	7 day	14 day
Control	90.5 ± 0.7^b,A^	103.6 ± 0.6^d,B^	107.4 ± 1.3^b,C^
HP-10 min	92.2 ± 3.8^b,A^	108.2 ± 1.0^c,C^	101.8 ± 1.2^cd,B^
HP-5 + 5 min	86.1 ± 1.5^c,A^	99.4 ± 1.4^e,B^	106.6 ± 0.5^b,C^
FTC-2	89.2 ± 1.5^bc,A^	109.9 ± 0.6^b,B^	115.2 ± 1.5^a,C^
FTC-4	88.3 ± 0.6^bc,A^	114.3 ± 0.9^a,C^	101.6 ± 0.8^d,B^
G12P	96.6 ± 1.1^a,A^	98.4 ± 1.2^e,A^	103.5 ± 0.4^c,B^
G24P	98.7 ± 0.7^a,A^	99.3 ± 0.9^e,A^	105.8 ± 0.6^b,B^

Values are means ± standard deviations (n = 3). Within columns, means followed by different letters are significantly different (*p* < 0.05). Within rows, means followed by different capitals are significantly different (*p* < 0.05).

During storage, CD content of the control increased by 13.1 μmol/100 g in the first 7 days and by only 3.8 μmol/100 g in the next 7 days. The slowdown in growth of CD content was also observed in HP-5 + 5 min and FTC-2, and even decreases in CD content were shown in HP-10 min and FTC-4 from 7th day to 14th day. FTC treated BR showed substantially higher CD content than other samples after 7 days of storage, especially FTC-4. In contrast, GP treated BR had higher CD content immediately after treatment, but showed better primary oxidation stability. Stored for 7 days, CD contents of G12P and G24P showed no significant change, and were substantially lower than those of other BR. Substantial increase in CD content (by 6.9–7.1 μmol/100 g) were observed after 14 days of accelerated storage.

### TBA value

In the secondary oxidation stage, lipid are degraded to volatile short-chain aldehydes and ketones such as MDA, resulting in off-flavor. The results of TBA value (MDA content) were listed in Table 3. After treatments, FTC treated BR showed the highest TBA value, especially FTC-2 (5.40 nmol/g), indicating a greater degree of secondary oxidation of lipids. HP-10 min contained 3.62 nmol/g of MDA, which was substantially lower than that of control (4.74 nmol/g). The TBA value of HP-5 + 5 min was 1.13 nmol/g higher than HP-10 min. GP treatment also decreased the MDA content to 3.53 nmol/g (G12P) and 2.97 nmol/g (G24P).

**Table 3:** 2-Thiobarbituric acid value (nmol/g) of brown rice processed with different treatments during storage.

Samples	Storage		
	0 day	7 day	14 day
Control	4.74 ± 0.09^c,B^	3.92 ± 0.06^f,A^	6.72 ± 0.08^a,C^
HP-10 min	3.62 ± 0.06^d,A^	4.79 ± 0.06^d,B^	5.61 ± 0.06^c,C^
HP-5 + 5 min	4.75 ± 0.06^c,A^	5.25 ± 0.04^c,B^	6.19 ± 0.04^b,C^
FTC-2	5.40 ± 0.09^a,A^	5.67 ± 0.06^a,B^	5.73 ± 0.02^c,B^
FTC-4	5.21 ± 0.13^b,A^	5.39 ± 0.08^b,A^	5.19 ± 0.15^d,A^
G12P	3.53 ± 0.11^d,A^	4.66 ± 0.10^e,B^	5.55 ± 0.13^c,C^
G24P	2.97 ± 0.06^e,A^	5.57 ± 0.10^a,C^	5.20 ± 0.17^d,B^

Values are means ± standard deviations (n = 3). Within columns, means followed by different letters are significantly different (*p* < 0.05). Within rows, means followed by different capitals are significantly different (*p* < 0.05).

During storage, MDA content in all BR samples showed different trends. The TBA value of control decreased by 0.82 nmol/g after 7 days, followed by a substantial increase by 71.4%. HP treated BR showed continuously promotion in MDA content, up to 5.61 nmol/g (HP-10 min) and 6.19 nmol/g (HP-5 + 5 min) after 14 days. Similar variation trend of TBA value was also observed in G12P, while G24P showed slightly decline in TBA value from 7th day (5.57 nmol/g) to 14th day (5.20 nmol/g). The TBA value of FTC-2 increased slightly by 5% in first 7 days, and then kept stable with the extension of storage time, while FTC-4 showed no significant change in TBA value during 14 days of storage. Overall, MDA content of all treated BR remained relatively stable in the later stage of accelerated storage and were substantially lower than that of control.

## Discussion

The results of fatty acids release after in-vitro digestion were mainly attributed to the changes of fatty acids content and digestive behavior of BR induced by all treatments. HP and FTC treatments increased free fatty acids content of BR, causing digested BR to release more fatty acids. The further generation of free fatty acids could be due to enhanced enzymatic hydrolysis of triglycerides. For HP treatment, similar increase in fat acidity was also observed in peony seeds oil^20^. The pressure (200 MPa) used in this study was reported to enhance lipase activity by 18%^21^. The effects of HP treatment on fat acidity depend on food matrix and treating conditions including pressure level, holding time, etc.^22^. HP-5 + 5 min showed substantially higher fat acidity than HP-10 min, meaning that HP treatment for 5 + 5 min could enhance enzymatic hydrolysis better than HP 10 min. This result was also helpful to explain the higher amount of released fatty acids in HP-5 + 5 min after digestion.

For FTC treatment, Qi et al.^23^ also found the free fatty acids value of ovine meat increased by 32–73% after 5–15 cycles of freeze–thaw. Ice crystallization destroyed cell membranes/walls during freezing at −20 °C, and then more lipase dripped out to hydrolyze lipids into free fatty acids during the subsequent processing of thawing and drying at 35–40 °C. There was no significant difference in fat acidity between HP-5 + 5 min and FTC treated BR, but the latter (especially FTC-4) released more fatty acids after digestion. This phenomenon could be explained by the differences in digestion. Lipids in BR mainly distribute in the indigestible rice bran layers. FTC treatment destructed bran layers because of ice expansion and this structural damage was severer as more cycles of freeze-thaw^1^. Finally, it enhanced the contact between lipids and digestive solution.

GP treatments decreased the fatty acids content of BR, and this decrease was likely to occur during parboiling processing. Wang et al.^24^ reported that damp heat treatment by steaming, same as the method we applied in this study, continuously decreased the fat acidity value of rice bran with the extension of processing time from 20 to 120 s. In this study, pre-germinated BR was parboiled by steam for 600 s, so it could be inferred that the thermal degradation of free fatty acids was more persistent and obvious in GP treated BR, resulting in a lower fat acidity value than the control. Although GP treated BR had the lowest content of free fatty acids, these samples still released the most fatty acids after digestion, indicating that more lipids were involved in the digestion process. The starch digestibility of BR might play a nonnegligible role in lipids digestion because this study focused on whole BR samples, of which starch accounts for at least 80%. The starch in the outmost layer of the endosperm is closely connected to the rice bran layers. When this starch is digested quickly, the lipids in bran layers are also more easily exposed to digestive solution. It had been proven that GP treated BR showed much better digestibility of starch than BR treated with HP and FTC^1^. Moreover, this previous study^1^ also found that G12P has thinner bran layers than G24P. Therefore, the digestive solution could more easily access the lipids stored in the bran layers of G12P, which helped to explain the largest amount of fatty acids released after digestion.

The increase of the total release of fatty acids was mainly reflected in released SFA, while released USFA showed different results. This might be due to that USFA are more prone to oxidative degradation than SFA. The higher CD content of GP treated BR confirmed the primary oxidation of lipids, which might occur during parboiling. Several previous study reported that heat treatment induced increase in CD content of lard^25^, palm olein^26^, vegetable oils^27^. Lipids are sensitive to thermal oxidation at high temperature to produce peroxides.

By contrast, the two nonthermal treatments did not increase the CD content of BR. However, FTC treatment increased TBA value of BR. CD is the intermediate product of oxidative rancidity of lipid. This result indicated that CD in BR was being further oxidized at the same time as it was produced during FTC treatment. Ali et al.^28^ also found that the lipid oxidation of chicken was intensified after FTC treatment. As the internal cause, ice crystallization damaged the cell membranes^29^. On the one hand, lipoxygenase was more likely to drip out to contact with lipids. On the other hand, lipids in cell membrane were more sensitive to oxidation than these in the triglyceride fraction^30^. As the external cause, more oxygen could permeate into bran layers during drying because FTC treatment had improved permeability of BR^1^.

After HP treatment, HP-5 + 5 min showed no significant change in MDA content, while the decrease was observed in HP-10 min. It was reported that soaking reduced the MDA content in BR because of the water-solubility of MDA, and HP treatment could enhance the leaching of MDA^11^. In the same way, the loss of MDA in GP treated BR might also occur during germination soaked in water, especially for 24 h. In spite of the leaching of MDA, HP-5 + 5 min still maintained the same MDA content as untreated BR, indicating that HP treatment also stimulated the secondary oxidation of lipids to generate MDA. HP-5 + 5 min showed substantially higher TBA value than HP-10 min, possibly due to one more decompression process. During the rapid release of high pressure, there is a transient pressure difference between inside and outside the cell, leading to the damage of cell membrane.

During accelerated storage, all BR samples showed various degrees of hydrolytic and oxidative rancidity. The fat acidity of FTC treated BR were not only higher than that of the control after treatment immediately, but also kept the highest value during storage. Liang et al.^31^ found that the more oxygen BR was exposed to during storage, the higher its fat acidity value. FTC treatment destroyed the bran layers structure of BR and promoted the permeability of bran layers^1^, making it easier for oxygen to enter into BR. On the contrary, although HP treatment promoted hydrolysis of lipids immediately, it enhanced the storage stability of free fatty acids in BR. It was in accordance with the results in previous article^11^. This result could be attributed to microbial inactivation induced by HP. Free fatty acids in BR are formed partly from lipid hydrolysis by endogenous enzymes and partly from lipid degradation by microorganisms, especially mould^32^. Better storage stability of fat acidity were observed in GP treated BR. Various heating pretreatments had been proven to inhibit the accumulation of free fatty acids in BR during storage^33,34^. On the one hand, parboiling could denature part of lipase originally present in BR, and on the other hand, it could inactivate some microorganisms, thus reducing the synthesis of lipase during storage.

On the whole, more CD accumulated in all BR samples after storage. After 7 days, FTC treated BR suffered from severer primary oxidative rancidity. The immediate cause was that freezing caused more lipoxygenase to be released from damaged cell and more oxygen to permeate the damaged bran layers. The indirect cause was that FTC treatment increased the free fatty acids content as shown in Fig. 1. Compared with the fatty acid moieties of glycerol esters, free fatty acids were more sensitive substrates for oxidation^35^. Subsequently, the slowdown and decline of CD content were observed, which were also shown in control and HP treated BR. Liu et al.^36^ also reported the similar variation trend in stored BR. The lipid primary oxidation products (such as CD) are the substrates of the secondary oxidation. Therefore, this result indicated that primary oxidation was dominant during the first 7 days, and then further secondary oxidation enhanced with prolonged storage time. The changes of MDA content in untreated and HP treated BR confirmed this conclusion. However, the TBA value of FTC treated BR barely changed during storage. Considering that FTC treated BR showed highest content of CD and MDA at certain stage, we speculated that oxidative rancidity occurred continuously and the MDA content was in a dynamic equilibrium state during storage. Owing to higher permeability of FTC treated BR (especially FTC-4) than that of other BR samples^1^, the MDA produced by secondary oxidation of lipids was more likely to volatilize into the external environment. There might be a balance between generation and volatilization of MDA. Consequently, it is recommended to store FTC treated BR with proper oxygen isolation technologies.

HP and GP treatment enhanced the oxidation stability of BR lipids, especially GP treatment. Lun^37^ found that MDA content in rice was positively correlated with microbial activity (*p* < 0.01) during storage. The inhibition of oxidative rancidity could be due to the sterilization of HP and GP treatment. Besides, the heat treatment (parboiling) could inactivate lipoxygenase to improve the oxidation stability of BR lipids. Similar results were also reported in preheated walnut paste^38^ and oil extracted from rice bran^39^. In addition, HP treatment was found to promote the contents of γ-Oryzanol and vitamin E, which are antioxidant compounds, in BR^40^.

## Conclusion

This study evaluated the lipid digestibility and rancidity of BR treated with different treatments. After in vitro digestion of BR, the released free fatty acids were mainly palmitic acid, stearic acid, oleic acid and linoleic acid. All treatments promoted the release of fatty acids (especially palmitic acid and stearic acid), and HP < FTC < GP with respect to the order of increase. FTC treatment exacerbated the hydrolytic rancidity in BR during whole storage, while GP treatment improved the stability of hydrolytic rancidity. HP treatment caused severer hydrolytic rancidity immediately, but inhibited further intensification under accelerated storage conditions. As for oxidative rancidity in BR, HP treatment had inhibitory effect, promoting storage stability. GP treated BR showed higher CD content and lower MDA content immediately after treatment, and better lipid oxidation stability during storage. FTC treated BR suffered from severe oxidative rancidity in the early stage of storage. In summary, GP treated BR were regarded as better products to supplement the extra intake of fatty acids. BR treated with HP and GP treatments showed better lipid stability, while FTC treated BR products were extremely suggested to be stored under anaerobic conditions.

## Data Availability

The datasets generated during and/or analyzed during the current study are available from the corresponding author on reasonable request.
